# What is context anyway? Reflections on the experience of the Reproductive Health Working Group in the Arab Countries and Turkey

**DOI:** 10.1186/s12978-025-01986-3

**Published:** 2025-05-31

**Authors:** Livia Wick, Hanan Abdul Rahim, Jocelyn DeJong, Weeam Hammoudeh, Aysecan Terzioglu, Yesim Yasin

**Affiliations:** 1 American University of Beirut, Beirut, Lebanon; 2 Qatar University, Doha, Qatar; 3 Birzeit University, Birzeit, Palestine; 4 Sabanci University, Istanbul, Turkey; 5 Acibadem University, Istanbul, Turkey

**Keywords:** Context, Reproductive Health, Gender, Arab countries, Turkey, War, Crises, Research Network

## Abstract

**Background:**

Contextualizing research involves making connections to the broader environment to understand relationships and relevance. This commentary zooms in on the question of how context matters to knowledge production by reflecting on the experience of the Reproductive Health Working Group in Arab Countries and Turkey (RHWG).

**Summary:**

In a region such as the Middle East and North Africa that is living through ongoing wars and crises, the network has paid attention to context in multiple ways: as an object of study, as a way to structure the network itself and as a way to conceptualize research support. From its very beginning in 1988, RHWG focused on the importance of social context to understand reproductive health issues. With time, the network became a capacity-building body supporting researchers working on gender and health in situations in which the context itself (the field or the site of empirical data collection) was being destroyed by crises and wars. Furthermore, context structures network activities, in particular the research workshop. The research workshop brings together scholars from the region in a supportive space to share their work thereby reducing isolation caused by wars and crises. In addition, context is an organizing principle and object of study in and of itself at the workshops. Discussions and interpretations of the research papers involve contextualizing them, that is making connections to relations in its surroundings.

**Conclusion:**

We have learned over the years that contextualizing research projects is not simply about putting together a historical and social story alongside the work on reproductive health. Contextualizing means thinking about what the concept of context does. In addition, we have learned that to do contextualized research we need to overcome the isolation of researchers created by wars and borders.

## Résumé

**Contexte:**

La contextualisation de la recherche implique d’établir des liens avec l’environnement plus large pour comprendre les relations et la pertinence. Ce commentaire porte sur l’importance du contexte pour la production de connaissances en réfléchissant à l’expérience du Groupe de travail sur la santé de la reproduction (RHWG) dans les pays arabes et en Turquie.

**Sommaire:**

Dans une région comme le Moyen-Orient et l’Afrique du Nord qui traverse des guerres et des crises continues, le réseau a porté attention au contexte de multiples façons : comme objet d’étude, comme moyen de structurer le réseau lui-même et comme moyen de conceptualiser le soutien à la recherche. Dès sa création en 1988, le RHWG s’est concentré sur l’importance du contexte social pour comprendre les questions portant sur la santé de la reproduction. Avec le temps, le réseau est devenu un organisme de renforcement des capacités soutenant les chercheurs travaillant sur l’égalité entre les sexes et la santé dans des situations où le contexte lui-même (le terrain ou le site de collecte de données empiriques) était détruit par des crises et des guerres. En outre, le contexte structure les activités du réseau, en particulier l’atelier de recherche, qui rassemble des chercheurs de la région dans un espace de soutien pour partager leurs travaux, réduisant ainsi l’isolement causé par les guerres et les crises. Le contexte est aussi un principe d’organisation et un objet d’étude en soi lors des ateliers. Les discussions et les interprétations des articles de recherche impliquent de les contextualiser, c’est-à-dire d’établir des liens avec les relations dans leur environnement.

**Conclusion:**

Nous avons appris au fil des ans que la contextualisation des projets de recherche ne consiste pas simplement à réunir un récit historique et social parallèlement au travail sur la santé de la reproduction. Contextualiser signifie réfléchir à ce que fait le concept de contexte. De plus, nous avons appris que pour effectuer des recherches contextualisées, nous devons surmonter l’isolement des chercheurs créé par les guerres et les frontières.

## Background

The special issue’s title “Context Matters” connects closely with the work of the Reproductive Health Working Group in the Arab Countries and Turkey (RHWG). A focus on context has been essential to the network’s existence and development over the years. In a region such as the Middle East and North Africa that is living through ongoing wars and crises, the concept of “context” is central to the research process in tangible ways. The network pays attention to context in multiple avenues: as an object of study, as a way to structure the network itself and as a way to conceptualize research support.

In 1988, Huda Zurayk, professor and researcher in public health at the Population Council, founded the RHWG in Cairo, Egypt. She and her founding colleagues envisioned the network as a regional initiative to encourage and to bring together research on women’s health in the social context of the Arab Countries and Turkey. It is noteworthy that the network was formed well before the 1994 United Nations (UN) International conference on Population and Development in Cairo when the term ‘reproductive health’ was first internationally endorsed, highlighting its critical and pioneering work. It is also noteworthy that “the social context” was essential to the ways the network first described itself [1–3]. Over the years, the RHWG developed into a capacity-building network of scholars that provides support to researchers working on reproductive health, loosely construed, and who are mainly resident in countries of the Arab World and Turkey. Researchers active in the network conduct research about gender and health in different contexts and fields, including public health, anthropology, sociology, medicine and midwifery.

What does it mean to contextualize research? What does it mean that RHWG conceptualizes itself around the concept of context? Who defines ‘context’ and from what disciplinary vantage point(s)? The practices of the network illustrate three dimensions of the concept of context.

## Context as an object of study

Researchers in gender studies and reproductive health in the Middle East are faced with a dilemma in terms of pursuing research and contextualizing it. The region has seen tremendous improvements in important reproductive health indices, such as a decrease in maternal mortality ratios, increased life expectancy and increased literacy rates for women and men [4–6]. These significant changes are at least partly due to the construction of modern public health and education systems based on the welfare state model. Since the late 1970s, scholars from various disciplines have questioned the positivist history of the construction of the postcolonial state and of the modernization of societies and have noted the ways it has erased other, maybe pre-modern ways of living [7]. Nevertheless, there is general agreement, on the basis of reproductive health research, that there has been improvement in demographic and health indices.

However, the Middle East continues to be the site of wars (including civil wars, proxy wars and settler colonial wars, and a mix of each kind, involving countries and global powers from the region and outside of it), political crises and natural disasters. Wars, crises and disasters are accompanied by destruction and tremendous suffering and loss. Thus, the context in which researchers are collecting empirical data (in social sciences, this is referred to as ‘the field’) has become one where health systems are destroyed and where people experience instability. It is also a context where demographic indices of reproductive health, education and life expectancy have plummeted. How can researchers pose research questions relevant to contexts of destruction? What have we learned from researching experiences of suffering, lament and loss? These are some of the questions and dilemmas that researchers at the RHWG have posed and grappled with over the years and have discussed at our regular workshops.

## Context structures the network activities

The question of context seeps through all the network’s activities, especially the research workshops. Organized approximately every two years, these gatherings are rich fora where members of the RWHG network present and workshop their research papers. Over the course of three decades, these workshops have provided a safe and supportive space for researchers to present work in progress and receive valuable feedback, sometimes as early in the research process as writing a proposal. Some network members have called the feedback they received at RHWG “constructive criticism”, a vital alternative to the competitive norms of global academia. In addition, researchers of the RHWG share experiences of conducting research in various national contexts, including some that are characterized by political or military conflicts, militarized borders, and restrictions on mobility. Thus, attention to context is essential to the research workshops—both in how it is structured and implemented.

Furthermore, RHWG researchers often share experiences of the challenges of doing research in institutional cultures that limit the work and careers of women researchers. These can be either in the formal format of sharing a paper about these challenges or in the informal—and highly valuable—setting of ‘corridor talk’. For example, many women researchers in the network have caring responsibilities which they perform in the midst of uncertainty and conflict. Thus, context for these women as researchers, caregivers and citizens in a volatile setting is important at the level of national politics, academic institutions and the family. Hence, the RHWG research workshops are intentionally designed to help researchers overcome isolation created by these different kinds of institutional, national and socio-cultural contexts.

At the research workshops, RHWG brings a discussion contextualized in the region in dialogue with global debates about reproductive health. The workshops provide an important contribution to reproductive health because they are a forum where researchers consider international debates and concepts (often feminist ones) and explore how these resonate or do not and play out in the context of the region. Thus, one of the central organizing concepts of the workshops over the years has been that of context.

At these workshops, context is an organizing principle and an object of study in and of itself. Part of the discussion and interpretation of the research papers and presentations involves making connections (and by implication, disconnections). To contextualize is to connect in a particular way; to connect to relations in its surroundings. The research workshops have been focused around the research object and subjects (childbirth, adolescent health, refugees’ access to health, workers’ health conditions etc.) and the interpretive and analytic strategies of making specific kinds of connections to their context and surroundings.

The geographic scope of the RHWG encompassing both Arab countries (often brought together in scholarship through the designation of the Arab World) and Turkey has been one basis for the emphasis on the study of context. This scope has pushed the boundaries of the definition of context beyond the typical boundaries of ethno-nationalist states and beyond simplistic conceptions of Arab culture or Turkish culture. Over many years, the design and execution of the RHWG research workshops has acknowledged, debated and responded to the contextual structures and practices that both define the issues of interest, alongside how they can be meaningfully addressed through research.

## Context as way to conceptualize research support

One aspect of the context that many countries of the region share is that of wars and disasters. The context of wars and disasters not only threatens and destroys the field or site where researchers collect empirical data (be they documents, archives, interviews, surveys and observations) but also threatens the institutions and universities where researchers are located. Recurrently, RHWG has had members who are displaced by these wars and crises. In 2023, for example, Sudanese members of RHWG were displaced to Cairo, Egypt. In 2024, Palestinian Gazan members were displaced to Cairo as well. They had to leave their work, find new work and sources of income and redefine their research in a new context. In earlier years, some RHWG members in Syria and others in Turkey were displaced because of wars, political crises and persecution. In some of these cases, RHWG was able to provide support to displaced scholars through its seed grants program, providing small funding opportunities to these scholars who had to begin new research projects in host countries. In contexts of long-term war, like in Palestine, RHWG has consistently provided outlets for discussing work and giving feedback, support and reducing isolation. Indeed, the RHWG workshops provided a crucial space for these researchers to share experiences and to push their research projects forward despite the constraints they faced. Thus, the RHWG has come to conceptualize and structure research support in terms of these contexts and has identified displaced scholars as an important area of the network’s work.

Discussions at RHWG research workshops allow researchers to bring out the similarities and differences between crises and wars in different contexts and between doing research in these different contexts. The strength of the discussions is that they permit researchers to avoid exceptionalism and connect various sites within the boundaries of ethno-nationalist states, the region and outside of it, without losing attention to detail. This is possible because of the trans-national constituency of the network and the in-depth contextual knowledge and grounding members have. Recent work often criticizes scholarship produced during colonial times in the nineteenth and early twentieth century as having failed to write about the colonial context in which the scholarship was produced. However, the national contexts in which scholarship is produced today has not received the same attention. Academic attention often focuses on Turkish studies or Arab studies, isolating them to a specific national space instead of linking places that have connected histories and societies. RHWG workshops provide a space to transcend scholarly attention to national contexts and make connections across ethno-linguistic and national boundaries.

Contexts of wars and crises are accompanied by deep divisions in society as well as by political polarization. The RHWG has been able to navigate such divisions by constructing spaces, such as the workshops, where researchers can express differing political perspectives. They are a safe and supportive space. The RHWG members are multi-generational and, through research workshops, scholars from different generations advise, mentor and support others. This has been the core of how the workshops function and a core mission of the RHWG. Thus, in all these different ways, the research workshops make context central to its discussions but transcend some major political rifts by creating a conducive and nurturing space for researchers.

## Conclusions

To go back to the opening question: what is context? What does it mean to contextualize? We have learned over the years that contextualizing research projects is not simply about putting together a historical and social story alongside the work on reproductive health. We have learned that contextualizing means thinking about what the concept of context does, who it includes and who it excludes and how the concepts are different because of the context. Thus, the dynamic context itself shapes the kinds of research questions being asked, and is not just the setting of the research. We have learned that to do contextualized research we need to overcome the isolation of researchers created by wars and borders, bring them together and transcend the political rifts in societies by bringing different perspectives into discussion. Finally, we have learned that in order to be effective in a context of wars and crises, context needs to inform the mission and structure of the RHWG by shaping research support and research workshop discussions. In the end, context does matter, and is at the core of the mission and everyday practices of the RHWG.

## Data Availability

Not applicable.
